# A Test of the ‘Genetic Rescue’ Technique Using Bottlenecked Donor Populations of *Drosophila melanogaster*

**DOI:** 10.1371/journal.pone.0043113

**Published:** 2012-08-13

**Authors:** Sol Heber, James V. Briskie, Luis A. Apiolaza

**Affiliations:** 1 School of Biological Sciences, University of Canterbury, Christchurch, New Zealand; 2 School of Forestry, University of Canterbury, Private Bag, Christchurch, New Zealand; Alexander Flemming Biomedical Sciences Research Center, Greece

## Abstract

We produced replicated experimental lines of inbred fruit flies *Drosophila melanogaster* to test the effects of crossing different bottlenecked populations as a method of ‘genetic rescue’ for endangered species lacking outbred donor populations. Two strains differing in the origin of the founders were maintained as isolated populations in a laboratory environment. After two generations of controlled full-sib matings, the resulting inbred fruit flies had significantly reduced breeding success and survival rates. However, crosses between the two bottlenecked strains reversed the effects of inbreeding and led to increases in breeding success and survival that persisted into the second generation of hybrid offspring. In contrast, crosses within each strain (but between different replicate lines) resulted in only slight improvements in some fitness components, and this positive trend was reversed in the second generation. This experiment highlights the potential value of translocations between different inbred populations of endangered species as a tool to mitigate the negative effects of inbreeding, but this benefit may depend upon the origin of the populations. Our results also confirm the importance of maintaining adequate levels of genetic variation within populations and that severely bottlenecked populations should not be discounted as possible donors in genetic rescue programs for endangered species.

## Introduction

Anthropogenic influences such as habitat loss and fragmentation, the introduction of exotic predators, excessive hunting, and pollution have forced many species through severe population bottlenecks. Decreased effective population size during a bottleneck can lead to increased inbreeding and the loss of genetic diversity, which both adversely affect population viability [1]–[6]. The translocation of outbred individuals into bottlenecked populations has been shown to mitigate the negative effects of inbreeding and to restore genetic variability (e.g. [7], [8], [9]). For example, the introduction of outbred individuals led to a rapid increase in the fitness of wild inbred populations of both greater prairie chickens (*Tympanuchus cupido*, [7]) and European adders (*Vipera berus*, [10]). Consequently, the ‘genetic rescue’ of endangered animals through the translocation of outbred individuals has become more frequent in recent years [11]–[13].

The use of genetic rescue as a management tool depends on the availability of suitable outbred donor populations. However, for many endangered species there are no outbred populations left to act as a donor. Instead, many endangered species survive only as a series of small, fragmented populations, with each likely subject to some loss of genetic variation and increased levels of inbreeding. Theoretical models suggest that by crossing individuals from one inbred population with those of a second inbred population, the severity of inbreeding depression should decrease in the hybrid offspring [14]. Such an effect might be expected if recessive deleterious alleles in one population become masked by alleles in the second population, and vice versa [15], [16]. Experiments with fruit flies (*Drosophila melanogaster*) and houseflies (*Musca domestica*) support the prediction that immigration of individuals into inbred lines can lead to rapid improvements in fitness traits such as viability, productivity and survival [17], [18] (see also [19]). In one of the few studies to use inbred donors in the genetic rescue of a wild animal, Fredrickson et al. [20] translocated inbred Mexican wolves (*Canis lupus baileyi*) to both captive and reintroduced populations of this species. As only three captive lineages of Mexican wolves survived from a total founding population of 7 animals, no outbred individuals were available as donors. Despite low levels of genetic variation and fixed deleterious alleles within each lineage, crosses between lines experienced increases in the proportion of live births, litter size, and survival of offspring [20].

Despite the apparent success of the genetic rescue technique using inbred donors in lab and field studies, the general effectiveness of using inbred individuals as donors is not clear, nor whether the suitability of inbred donors varies with their source. In some species, prospective donor populations may share a recent common ancestry with a recipient population (as is the case with many daughter populations created through the translocation of individuals to found new populations for conservation purposes), and may not be differentiated enough to introduce new genetic variation. Alternatively, a donor population may be so differentiated (as may be the case for two subspecies or geographically isolated populations) that it may lead to a deterioration of fitness traits, in a process termed outbreeding depression [13], [14], [16], [21]–[27]. The objective of this study was therefore to test whether the exchange of individuals between inbred populations reduces levels of inbreeding depression, and if the effectiveness of any change depends on the source of the donor population. To address this question, we conducted replicated experimental crossings within and between two artificially inbred strains of the fruit fly *Drosophila melanogaster* to test changes in the viability of the hybridised population.

## Materials and Methods

### Inbreeding Method

**Figure 1:**
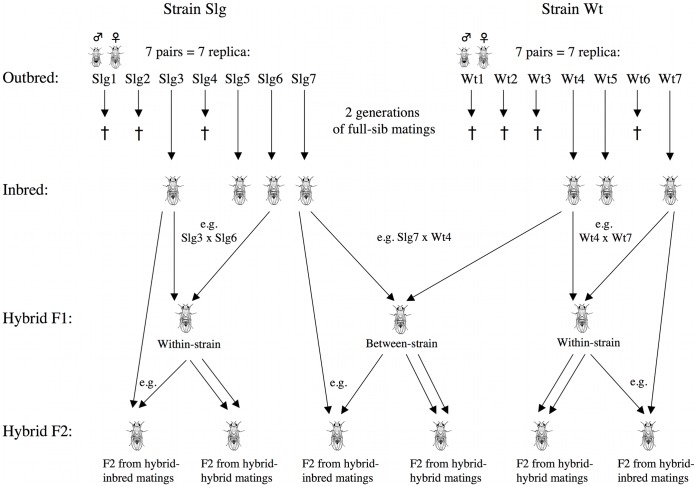
Diagram of the crossing experiment. Crosses (<$>\raster="rg1"<$>) identify replica lost during the process of inbreeding. In the between-strain F2 hybrids, offspring from hybrid-inbred matings resulted either from matings of F1 hybrids with Slg inbred or Wt inbred flies (only matings between F1 between-strain hybrids and Slg inbred flies are shown for simplicity). Pairs that did not lay any eggs are excluded from the calculations.

Two strains of *Drosophila melanogaster* originating from different parts of the world (Wild type Oregon-R, USA, and Slg14–15, Sweden) were used to create inbred lineages. Both source populations were maintained in cages supporting >500 individuals with overlapping generations. Despite the maintenance of large populations, stocks of fruit flies are known to lose genetic variation, with the degree of loss increasing with greater periods of time in captivity [28]. Although we did not measure levels of genetic variation in each population directly, high levels of fertility and survival among individuals suggest neither was suffering inbreeding depression. We then created replicate inbred lines within each strain through two generations of full-sib matings. From each line, offspring were collected as virgins, and one full-sib pair was randomly chosen as parents for the next generation. Each pair was housed in separate vials to prevent outbreeding (vials measured 75 mm×25 mm×25 mm). All eclosed young were removed twice a day to ensure virgins were used for the next generation. The inbreeding procedure was stopped after two generations, as both populations experienced problems with reproductive success and survival. Seven replica of full-sib pairs within each strain were started; however, three replica in the Slg14–15 strain and four replica in the Wild type Oregon-R strain were lost due to complete reproductive failure (see crosses in figure 1). The extinction of 7/14 (50%) of inbred lines is consistent with an expected increase in the risk of extinction with increased inbreeding [5].

All cultures were maintained on standard commercial medium (Formula 4–24 instant medium, blue, Carolina Biological Supply Company, North Carolina, USA) with a supplement of live yeast. The stocks were kept in an incubator at 25±1.0°C, and a 12∶12 hour light:dark photoperiodic cycle. The position of vials within the incubator was re-randomised on a regular basis.

### Crossing Experiments

Four inbred replica of the Slg14–15 strain (hereafter, Slg) and 3 inbred replica of the Wild type Oregon-R strain (hereafter, Wt) survived for 2 generations. The flies from these 7 lines (named Wt1, Wt2, Slg1, Slg2, etc.) were used for replicate crossing ( =  “genetic rescue”) experiments. To test the effects of hybridising individuals from inbred lines on the fitness of offspring, we paired inbred flies within each strain, but between different replica (e.g. Slg5 × Slg7, 20 pairs; e.g. Wt4 × Wt5, 27 pairs). The resulting offspring are referred to as F1 within-strain hybrids. This tested whether inbred populations founded from the same population could still act as genetic rescue donors. We also paired inbred flies from one strain with inbred flies from the other strain (e.g. Slg6 × Wt7; 27 pairs), to test the effects of hybridising inbred individuals from differing strains on the fitness of their offspring. These are termed F1 between-strain hybrids (Slg-Wt; see figure 1). With these crossings, we tested whether inbred populations founded from different source populations could act as genetic rescue donors.

To determine the persistence of fitness effects from hybridising inbred lines, a second generation of hybrids (F2) was bred within each F1 within-strain and between-strain hybrids. Within each of the three groups (Slg within-strain hybrids, Wt within-strain hybrids, and Slg-Wt between-strain hybrids), F1 hybrids were either paired with other F1 hybrids of the same group (hybrid-hybrid matings; Slg: 19 pairs, Wt: 29 pairs, Slg-Wt: 29 pairs), or with inbred flies of the same strain (hybrid-inbred matings; Slg: 23 pairs, Wt: 33 pairs), or of both strains in the case of the between-strain crosses (Slg-Wt: 30 pairs), resulting in six groups in the F2 hybrid generation (F2 hybrids resulting from either hybrid-hybrid or hybrid-inbred matings in each strain, Slg, Wt and Slg-Wt, respectively; see figure 1). Pairs from the original populations were used as controls (37 pairs). The final number of pairings in each group varied due to the death of some flies during the course of the experiment. Both reproductive success and survival are fitness measures vital to the persistence of populations. Breeding success and daily survival rates were therefore assessed for pairs and individuals in each group (inbred, F1 hybrid, F2 hybrid, and outbred).

### Breeding Success

Each pair was put into a clean vial with fresh medium and allowed to mate and oviposit for 96 h. Eggs were counted using a Wild Heerbrugg M3 stereomicroscope upon removal of the adults. Daily emergence of male and female adult progeny was counted twice a day until eclosion stopped. The total number of pupae and the number of not eclosed pupae were counted. In terms of absolute reproductive output, only the average number of eggs laid per pair is reported here, as the absolute numbers of pupae formed and adults eclosed depend on the number of eggs laid. The proportion of eggs that developed into pupae and pupae that developed into adults was calculated and used as measures of reproductive success. Pairs that did not lay any eggs were excluded from the calculations.

### Survival

Upon eclosion, flies were counted and sexed under CO_2_ anaesthesia. Males and females were then transferred to new same-sex vials with standard instant medium, with a total of 20 flies per vial. Vials were checked daily up to a maximum of 8 days to record the number of dead flies. Flies still alive after 8 days were censored in the analysis (see below) to account for the end of the observation period.

### Data Analyses

As measures of breeding success did not differ significantly between the Slg and Wt strains (Wilcoxon Signed Rank tests; number of eggs laid: p = 0.13, proportion of eggs that pupated: p = 0.61, proportion of pupae that eclosed: p = 0.12), the two strains were pooled to facilitate comparison with the between-strain hybrids. This resulted in two groups in the first generation (hybrid F1 within-strain (Slg and Wt) vs. hybrid F1 between-strain (Slg-Wt)). As mean trait values of reproductive success of hybrid-hybrid and hybrid-inbred pairs were not significantly different in both F2 within-strain and F2 between-strain crosses (all credible intervals include 1), we pooled this data, resulting in two groups of F2 hybrids (hybrid F2 within-strain and hybrid F2 between-strain). We therefore compared a total of six groups of varying inbreeding status (inbred, hybrid F1 within-strain, hybrid F2 within-strain, hybrid F1 between-strain, hybrid F2 between-strain, and outbred).

Data from the breeding experiments were fitted using an ‘animal model’ [29], which is a generalised linear mixed model that expresses phenotypic observations *y_i_* as a function of an additive genetic component *a_i_*. This model can accommodate the pedigree of the individuals through the use of a relatedness matrix. In addition to the genetic component, the model included a group effect with the six levels of inbreeding status. The number of eggs laid was analysed using a Gaussian distribution with an identity link function, while the proportion of eggs that pupated (pupae/eggs) and the proportion of pupae that eclosed (adults/pupae) were analysed using a binomial distribution with a logit link function. Inference for the animal model relied on a Bayesian framework, using Integrated Nested Laplace Approximations (INLA) to calculate the marginal posteriors for all parameters [30]. Marginal posteriors were summarised using the posterior mean and 95% credible intervals. Model fitting was performed using AnimalINLA [31], a package for the R statistical software system (see electronic Script S1 and Data S1 and S2 for the INLA analysis script and data files used in R, version 2.13.1; [32]).

As survival did not differ significantly between the Slg and Wt strains (Wilcoxon Signed Rank test, p = 0.72), the two strains were pooled to facilitate comparison with the between-strain hybrids. Data for each fruit fly in the survival trial consisted of the time (in days) until exit from the study (either by death or by censoring), a censoring indicator specifying whether an individual survived until the end of the experiment or not (0 = individual survived and died at an unknown time in the future, 1 = individual died during the course of the experiment), and the group (inbred, hybrid F1 within-strain, hybrid F2 within-strain, hybrid F1 between-strain, hybrid F2 between-strain, and outbred) that the individual belonged to as factor. We analysed the survival data using the *survreg* function within the “survival” package in the R statistical environment (version 2.13.1; [32], [33]). We first fitted a parametric model assuming constant hazard of death (exponential error distribution) with censoring (as a number of individuals died at an unknown time after the end of the experiment). We then compared the model to a parametric model based on the Weibull distribution (assumption of age-specific non-constant hazard), which was a significant improvement (p<0.0001). This model was simplified by pooling the survival rate of F2 offspring resulting from hybrid-hybrid matings with that of F2 offspring resulting from hybrid-inbred matings, as there was no significant difference between the survival rates in within-strain (Slg and Wt) and between-strain hybrids (Slg-Wt; p = 0.973 and p = 0.405, respectively). The simpler model was not significantly worse compared to the initial model (p = 0.08). We calculated effect sizes and adjusted p-values for the model estimates with the add-on R package “multcomp” [34]. Adjusted 95% confidence intervals (CIs) were computed by hand using the adjusted p-values (see electronic Script S2 and Data S3 for the survival analysis script and data file used in R). The use of p-values adjusted for the multiple comparisons, and hence of adjusted 95% confidence intervals, is justified due to the large sample size used in the survival trial (n = 4,226; [35]). Daily survival probability was calculated using a modified Mayfield method [36].

## Results

### Breeding Success

Inbreeding within each line lead to a significant reduction in the absolute numbers of eggs laid (table 1). The effect sizes of the pairwise contrasts for the average number of eggs laid, and their 95% credible intervals can be found in table 2. Egg number increased progressively from inbred pairs to the first and second generation of hybrid between-strain pairs and reached a maximum in outbred pairs (table 1). The increase observed in F1 between-strain hybrids was not significant compared to inbred individuals (credible intervals include zero; table 2). However, F2 between-strain hybrids exhibited significantly higher values than inbred flies and F1 between-strain hybrids (credible intervals do not include zero; table 2), and did not significantly differ from outbred pairs (i.e., the number of eggs laid was comparable to that in outbred flies). In contrast, the number of eggs laid in the hybrid F1 within-strain group was virtually identical to values recorded for inbred pairs (table 1). However, the number of eggs laid increased in the second generation of within-strain hybrids, and was significantly higher than in inbred flies, but significantly lower than in outbred flies, and thus had an intermediate status between inbred and outbred pairs. In other words, both within-strain and between-strain hybrids produced an increased number of eggs, but the increase was more pronounced in between-strain hybrids than in within-strain hybrids. Only F2 between-strain hybrids were comparable to outbred flies in terms of the absolute number of eggs laid (table 1).

**Table 1:** Mean breeding values and 95% credible intervals (CIs) for absolute numbers of eggs laid, proportion of eggs that pupated, and proportion of pupae that eclosed for inbred, hybrid, and outbred groups of *Drosophila melanogaster* (ws: within-strain crosses, bs: between-strain crosses).

Trait	Cross	N (pairs)	Mean	Lower 95% CI	Upper 95% CI
Eggs	Inbred	44	38.17	23.60	52.71
	Hybrid F1 (ws)	47	39.10	25.08	53.05
	Hybrid F1 (bs)	27	55.59	37.94	73.27
	Hybrid F2 (ws)	101	64.61	53.37	75.85
	Hybrid F2 (bs)	62	94.06	80.45	107.64
	Outbred	37	99.34	83.53	115.15
Pupae/eggs	Inbred	44	0.77	0.57	0.89
	Hybrid F1 (ws)	47	0.81	0.64	0.91
	Hybrid F1 (bs)	27	0.76	0.54	0.89
	Hybrid F2 (ws)	100	0.78	0.62	0.89
	Hybrid F2 (bs)	61	0.93	0.85	0.97
	Outbred	37	0.95	0.87	0.98
Adults/pupae	Inbred	44	0.94	0.91	0.97
	Hybrid F1 (ws)	47	0.98	0.96	0.99
	Hybrid F1 (bs)	27	0.99	0.97	0.99
	Hybrid F2 (ws)	101	0.96	0.94	0.98
	Hybrid F2 (bs)	62	0.97	0.96	0.98
	Outbred	37	0.98	0.96	0.99

Pairs that did not lay any eggs are excluded from the calculations.

**Table 2:** Pairwise contrasts (effect size and 95% credible intervals) for absolute numbers of eggs laid for inbred, hybrid, and outbred groups of *Drosophila melanogaster* (ws: within-strain crosses, bs: between-strain crosses).

Comparison	Effect size	Lower 95% CI	Upper 95% CI
Inbred - Hybrid F1 (ws)	−0.90	−22.62	20.82
Inbred - Hybrid F1 (bs)	−17.57	**−**42.92	7.80
Inbred - Hybrid F2 (ws)	**−26.40**	**−45.91**	**−6.91**
Inbred - Hybrid F2 (bs)	**−55.85**	**−77.46**	**−34.30**
Hybrid F1 (ws) - Hybrid F1 (bs)	−16.67	−41.55	8.24
Hybrid F1 (ws) - Hybrid F2 (ws)	**−25.50**	**−44.13**	**−6.94**
Hybrid F1 (bs) - Hybrid F2 (bs)	**−38.28**	**−62.88**	**−13.89**
Hybrid F2 (ws) - Hybrid F2 (bs)	**−29.45**	**−47.61**	**−11.22**
Inbred - Outbred	**−61.17**	**−84.78**	**−37.57**
Hybrid F1 (ws) - Outbred	**−60.27**	**−83.41**	**−37.12**
Hybrid F1 (bs) - Outbred	**−43.60**	**−70.18**	**−17.04**
Hybrid F2 (ws) - Outbred	**−34.77**	**−55.50**	**−14.05**
Hybrid F2 (bs) - Outbred	−5.32	−28.10	17.43

Differences are significant if the 95% CIs do not include 0 and are indicated in bold.

The proportion of eggs that developed into pupae in inbred flies decreased by 18% compared to outbred flies (table 1). In contrast, the proportion of pupae that hatched was relatively high in all groups, ranging between 94% in inbred flies and 98% in outbred flies (table 1). Effect sizes of the pairwise odds ratios (and 95% credible intervals) for the proportion of eggs that pupated and pupae that hatched are listed in table 3 (note that in odds ratios, differences are significant if the 95% credible intervals do not include 1, as opposed to pairwise contrasts, where differences are significant if the 95% CIs do not include 0).

**Table 3:** Pairwise odds ratios (effect size and 95% credible intervals) for proportion of eggs that pupated and proportion of pupae that eclosed for inbred, hybrid, and outbred groups of *Drosophila melanogaster* (ws: within-strain crosses, bs: between-strain crosses).

Trait	Comparison	Odds ratio	Lower 95% CI	Upper 95% CI
Pupae/eggs	Inbred - Hybrid F1 (ws)	0.98	0.19	3.02
	Inbred - Hybrid F1 (bs)	1.40	0.24	4.56
	Inbred - Hybrid F2 (ws)	1.14	0.26	3.24
	Inbred - Hybrid F2 (bs)	**0.32**	**0.07**	**0.93**
	Hybrid F1 (ws) - Hybrid F1 (bs)	1.86	0.29	6.28
	Hybrid F1 (ws) - Hybrid F2 (ws)	1.39	0.42	3.40
	Hybrid F1 (bs) - Hybrid F2 (bs)	**0.30**	**0.08**	**0.79**
	Hybrid F2 (ws) - Hybrid F2 (bs)	0.36	0.07	1.09
	Inbred - Outbred	**0.23**	**0.03**	**0.81**
	Hybrid F1 (ws) - Outbred	0.30	0.04	1.06
	Hybrid F1 (bs) - Outbred	**0.23**	**0.03**	**0.84**
	Hybrid F2 (ws) - Outbred	**0.24**	**0.04**	**0.81**
	Hybrid F2 (bs) - Outbred	0.89	0.13	3.13
Adults/pupae	Inbred - Hybrid F1 (ws)	**0.38**	**0.13**	**0.86**
	Inbred - Hybrid F1 (bs)	**0.27**	**0.08**	**0.65**
	Inbred - Hybrid F2 (ws)	0.69	0.29	1.38
	Inbred - Hybrid F2 (bs)	**0.49**	**0.20**	**0.98**
	Hybrid F1 (ws) - Hybrid F1 (bs)	0.82	0.22	2.13
	Hybrid F1 (ws) - Hybrid F2 (ws)	2.04	0.92	3.94
	Hybrid F1 (bs) - Hybrid F2 (bs)	2.12	0.83	4.48
	Hybrid F2 (ws) - Hybrid F2 (bs)	0.76	0.32	1.53
	Inbred - Outbred	**0.44**	**0.15**	**0.98**
	Hybrid F1 (ws) - Outbred	1.32	0.43	3.12
	Hybrid F1 (bs) - Outbred	1.97	0.56	5.00
	Hybrid F2 (ws) - Outbred	0.67	0.26	1.42
	Hybrid F2 (bs) - Outbred	0.97	0.35	2.13

In odds ratios, differences are significant if the 95% credible intervals do not include 1. Significant differences are highlighted in bold.

In between-strain hybrids, pupating success in the F1 generation was virtually identical to that found in inbred flies; however, there was a significant increase in pupating success in the F2 generation and levels were not significantly different to those recorded in outbred flies (tables 1 and 3). Pupae eclosing success increased significantly in both the F1 and F2 generations of between-strain hybrids and was comparable to eclosing levels in outbred flies.

In F1 within-strain hybrids, the proportion of eggs that pupated tended to increase compared to inbred flies, but this increase was not significant (however, it was also not significantly lower than in outbred flies). In the F2 generation of within-strain hybrids, the proportion of eggs that pupated decreased to levels observed in inbred flies. Similarly, eclosing success of pupae increased in the F1 generation of within-strain hybrids and was virtually identical to eclosing levels in outbred flies, but decreased in the F2 generation to levels that were intermediate between inbred and outbred flies (tables 1 and 3).

### Survival

Daily mortality probabilities and results of the survival analyses (effect size of the pairwise comparisons, adjusted 95% confidence intervals and adjusted p-values) are summarised in table 4. In the F1 generation of hybrids, both within- and between-strain hybrids experienced a significant increase in survival probability, with the effect being more pronounced in between-strain hybrids (table 4). There was a slight reduction in survival probability from the F1 to the F2 generation of between-strain hybrids, but it was not significantly different from survival probability in outbred individuals. The positive effect seen in F1 within-strain hybrids, however, did not persist into the F2 generation, where survival was reduced to levels observed in inbred flies (table 4).

**Table 4:** Pairwise comparisons of mortality probabilities (as calculated using the Mayfield method), effect size and confidence intervals for the effect size (estimated using the *survreg* function) of *D. melanogaster* groups of varying level of inbreeding (ws: within-strain crosses, bs: between-strain crosses).

	Group	N	Daily mortality probability (%)	Effect size	Lower 95% CI (adjusted)	Upper 95% CI (adjusted)	Adjusted p-value
(1)	Inbred	332	5.8				
	Hybrid F1 (ws)	430	1.4	−0.785	−1.006	−0.563	<0.001***
(2)	Inbred	332	5.8				
	Hybrid F1 (bs)	405	0.2	−1.779	−2.353	−1.205	<0.001***
(3)	Inbred	332	5.8				
	Hybrid F2 (ws)	430	4.9	−0.161	−0.328	0.007	0.061
(4)	Inbred	332	5.8				
	Hybrid F2 (bs)	966	1.2	−0.762	−0.942	−0.582	<0.001***
(5)	Hybrid F1 (ws)	430	1.4				
	Hybrid F1 (bs)	405	0.2	−0.994	−1.689	−0.299	0.005**
(6)	Hybrid F1 (ws)	430	1.4				
	Hybrid F2 (ws)	1,586	4.9	0.624	0.420	0.828	<0.001***
(7)	Hybrid F1 (bs)	430	0.2				
	Hybrid F2 (bs)	966	1.2	1.017	0.355	1.680	0.003**
(8)	Hybrid F2 (ws)	1,586	4.9				
	Hybrid F2 (bs)	966	1.2	−0.601	−0.752	−0.450	<0.001***
(9)	Inbred	332	5.8				
	Outbred	507	2.0	−0.559	−0.742	−0.376	<0.001***
(10)	Hybrid F1 (ws)	430	1.4				
	Outbred	507	2.0	0.226	−0.229	0.680	0.33
(11)	Hybrid F1 (bs)	405	0.2				
	Outbred	507	2.0	1.220	0.589	1.851	<0.001***
(12)	Hybrid F2 (ws)	1,586	4.9				
	Outbred	507	2.0	−0.398	−0.562	−0.234	<0.001***
(13)	Hybrid F2 (bs)	966	1.2				
	Outbred	507	2.0	0.203	−0.135	0.540	0.24

## Discussion

As expected, the severe bottlenecks we induced in the two strains of fruit flies and the subsequent forced inbreeding led to a decline in individual breeding success and survival. Even after only two generations of inbreeding, half of our lines went extinct through reproductive failure. However, subsequent crossings within each strain of inbred flies (but between different replicate lines) as well as crossings between the two strains resulted in significant increases in survival and some measures of reproductive success. The positive effects of the crossing experiments were more pronounced in the between-strain hybrids, which increased even further in the F2 generation. Most importantly, between-strain hybrids exhibited a significant increase in both the absolute (number of eggs produced), and the relative reproductive output (proportion of eggs that developed into pupae, and proportion of pupae that eclosed). This was coupled with a marked increase in survival probability, which even exceeded the survival probabilities of outbred individuals. Our results thus support the potential value of genetic rescue as a management tool for endangered species that survive only as a series of fragmented and bottlenecked populations.

For within-strain crossings, the results were of mixed nature. In terms of absolute reproductive output (number of eggs laid), the first generation of within-strain hybrids showed no increase compared to inbred flies. Significant positive effects were, however, observed in the second generation of within-strain crossings. Relative reproductive output (the proportion of eggs that developed into pupae, and the proportion of pupae that eclosed) tended to increase in F1 within-strain hybrids, but in F2 individuals this positive trend was reversed to levels measured in inbred flies. Similarly, survival probabilities increased significantly in F1 within-strain hybrids compared to inbred flies, but this positive effect did not persist into the second generation. Nonetheless, the increase in number of eggs laid observed in the second generation of within-strain hybrids constitutes a fitness improvement compared to inbred flies. Whether the improvement in fitness is sufficient to warrant the implementation of genetic rescue between populations of endangered species stemming from the same source population is not clear and would first require determining whether such crosses would actually introduce any new genetic variation into the recipient populations. Given that a number of endangered species currently managed as discrete populations originated from the same single source population and yet show some genetic differentiation (e.g. black-footed ferret, *Mustela nigripes*; [37]), suggests further tests of the genetic rescue hypothesis using inbred populations descending from the same source would be worthwhile.

The relatively weak response we obtained to within-strain crosses contrasts with that obtained by Spielman & Frankham [18]. They found that even the introduction of a single immigrant into their fruit fly populations lead to an increase in fitness (as measured by a competitive index measure), even though the immigrants stemmed from the same base population. The difference with our results may be due to our use of lab strains while Spielman & Frankham [18] used a wild caught population as their source from which to start inbred lines. As lab stocks of *Drosophila* have lower level of genetic variation than their wild source populations [28], it is likely that more variation was present in their inbred lines than in our equivalent lines and thus our within-strain crosses injected relatively little new variation in the donor populations. For some endangered species, which survive as only two or three populations that stem from only as a single bottlenecked population, genetic variation is known be very low (e.g. black robin [38]), and the use of crosses in such species may be similar to our use of lab stocks. Although we cannot determine why our results differ from this earlier study, without direct estimates of genetic variation present in potential donor populations, the prudent course of action would be to use donors not recently sourced from the same population as the recipients.

Although we did not quantify genetic variability, we assumed that the two outbred strains used in this study had some degree of genetic differentiation, given that they stem from different parts of the world. Under this assumption, we would therefore expect the fruit fly lines resulting from the full-sib matings to have relatively large differences in their allelic composition between the two strains. In contrast, within-strain hybrids (i.e. crosses of inbred flies of the same strain) were expected to be genetically similar. Nevertheless, even replica of the same strain were unlikely to be genetically identical, and thus deleterious alleles could still be masked by crossing flies from different replica within each strain. The subsequent exposure of deleterious alleles in the second generation of within-strain hybrids could then cause the reversal of the positive effects seen in the F1 generation (e.g., observed reduction in survival of F2 within-strain hybrids to levels similar to inbred individuals). As the biggest improvements in reproductive success and survival were observed in between-strain hybrids (i.e., crosses of flies from two different strains that are likely to be genetically dissimilar), and these improvements persisted into the second generation, our observations are consistent with a concomitant increase in levels of genetic diversity in the hybrid offspring that persisted for at least two generations. However, an analysis of genotypes would be necessary to determine the exact mechanism for this fitness effect.

Outbreeding depression (reduced fitness in crosses between distantly related individuals) typically becomes apparent in the F2 generation of crosses, when the original parental gene combinations are split up by recombination processes such as chromosomal crossover and segregation, which can cause the disruption of extrinsic interactions between genes and the environment (e.g. of locally adapted gene complexes) or inherent interactions between genes [14], [21], [24], [25]. Interestingly, some traits in our study (e.g. the proportion of eggs that developed into pupae) showed no change in the first generation of between-strain hybrids, but positive effects appeared subsequently in the second generation. One possible explanation for this observation is that it is due to a maternal effect – inbred mothers could potentially be less effective in provisioning for eggs compared to hybrid or outbred mothers. Regardless, the increase in fitness in the F2 compared to F1 between-strain hybrids indicates that the original populations used in this study were not genetically differentiated enough to induce outbreeding depression in the between-strain hybrid offspring. Nonetheless, when planning a translocation of an endangered species it would be important to choose source populations that adaptively match the population of concern (e.g. adapted to similar environments) in order to avoid outbreeding depression (see also [13], [26], [27]).

As previously found in other studies (e.g. [39], [40]), fitness differences between inbred and outbred populations are primarily due to survivorship differences. Similarly, our crossing experiments revealed that the most important improvement in fitness was survival probability. Although breeding success in the first generation of hybridisation showed only slight improvement in both within- and between-strain hybrids, when coupled with increased survival, this meant that individuals in hybrid populations had more opportunities to reproduce, and this result therefore carries important implications for the persistence of threatened populations. Some previous studies of genetic rescue using outbred populations as a source have found increased reproductive success in the recipient populations (e.g. [7], [41]). However, even if genetic rescue using inbred populations does not induce a similar increase on a per breeding attempt basis, the technique may still provide management benefits if it increases adult survival and thus lifetime reproductive success.

The bottleneck that the two original populations used in this study were forced through (two generations of full-sib matings) was particularly severe. Although bottlenecks of this severity are unlikely to occur to the same extent in most natural populations, at least a few species have passed through bottlenecks that approach this level (e.g. black robin, *Petroica traversi*: 1 pair [42]; Mauritius kestrel, *Falco punctatus*: 1 pair [43]). For less critically endangered species, the effect of crossings on fitness might be less pronounced if they suffered a smaller loss of genetic variation from the outset, and it might therefore be valuable to test the effects of crossing individuals between populations of varying bottleneck size. Furthermore, the impact of hybridisation on fitness depends not solely on the level of parental genetic similarity, but also on effects of the environment. A way to investigate environmental effects in the laboratory could be to subject both inbred and hybrid individuals to changes in the environment, such as increases in temperature or salinity, or exposure to pathogens, and to test if the groups differ in their ability to respond to novel challenges (e.g. [44], [45]).

The use of lab animals such as *Drosophila* provides a convenient model for studying the consequences of inbreeding and testing potential methods to remediate the negative effects (e.g. [17], [18]). The objective of such studies, including ours, is to extrapolate these findings to more effectively manage populations of endangered organisms in the wild. However, caution is required in directly relating the results of lab studies to wild populations. The fruit flies in our study were provided with *ad libitum* food and a constant environment and, as far as we could tell, limited exposure to parasites and pathogens. Under such conditions, individuals with deleterious alleles may survive and reproduce that would otherwise not do so in the wild [46]. This could lead to an over-estimate of the benefits of donors to an inbred population, especially if the benefit is small, as it is likely to be the case with donors equivalent to our “within-strain” lines. Furthermore, the long-term consequences of genetic rescue of wild populations with inbred donors from differing source populations are not clear. Other workers have conducted longer-term studies of the effects of immigration into lab populations of insects [17], [19] and confirmed the benefits can persist for more than 3 generations. Whether a similar pattern is seen in wild populations, in which some of the introduced alleles may be removed by selection, needs to be determined.

With the increasing number of species around the world passing through severe population bottlenecks, the results of our study provide an empirical demonstration of the immediate fitness benefits of hybridising different inbred populations. Although restricted to a laboratory environment, our results are consistent with the purported benefits of an earlier attempt to use inbred individuals to rescue wild populations of the Mexican wolf [20]. Whether these effects also hold in other populations of wild animals, and whether such benefits persist for more than a few generations needs to be tested. Nevertheless, the use of severely bottlenecked populations as donors to preserve even the most critically endangered species should not be disregarded in view of the potential benefits and the current rapid increase in the number of species that survive only as small and isolated populations vulnerable to both demographic and genetic stochasticity.

## Supporting Information

Script S1 **INLA analysis script used in R.**
(R) Click here for additional data file.

Script S2 **Survival analysis script used in R.**
(R) Click here for additional data file.

Data S1 **Breeding data used in INLA analysis script.**
(CSV)

Data S2 **Pedigree data used in INLA analysis script.**
(CSV)

Data S3 **Survival data used in survival script.**
(TXT)
